# An experimental study on coflow diffusion combustion in a pellet-packed bed with different bed lengths

**DOI:** 10.1098/rsos.172027

**Published:** 2018-08-22

**Authors:** Junrui Shi, Yang Liu, Yongqi Liu, Mingming Mao, Yongfang Xia, Rui Ma, Youning Xu

**Affiliations:** 1 School of Transportation and Vehicle Engineering, Shandong University of Technology, Zibo, Shandong 255049, People's Republic of China; 2 Key Laboratory of Clean Combustion for Electric Generation and Heating Technology of Liaoning, Shenyang Institute of Engineering, Shenyang, Liaoning 110136, People's Republic of China

**Keywords:** diffusion flame, flame shape, flame height, oscillation, bed length

## Abstract

In experimental investigations on axial symmetry, over-ventilated CH_4_/air diffusion combustion in a packed bed is executed to study the height, shape and stability of the flame. The combustor is a quartz tube packed with alumina pellets in which a cylindrical fuel stream is surrounded by a coflow air nozzle. The results show that the bed length and pellet diameter have a significant influence on the flame properties. In general, the flame above the pellet surface has axial symmetry, and its shape and colour are similar to those of a conventional diffusion flame when the bed length is smaller. The colour of the flame front varies with the bed length. The changed colour indicates an increased flame front temperature and that the combustion regime above the bed surface may change from non-premixed combustion to partially premixed combustion or even premixed combustion owing to the mix and dispersion effect in the packed bed. In addition, multiple flame behaviours, such as an inclined flame front, isolated reaction zone and oscillatory motion followed by a pulsating sound with a few hertz in a packed bed, are observed experimentally. The possible reasons for these phenomena are discussed.

## 1. Introduction

In recent decades, filtration combustion research has mainly focused on premixed combustion in porous media, and some significant developments have occurred. For example, the filtration combustion wave speed, flame thickness, lean flammability limit and flame stability can be predicted well numerically or theoretically [1–10] (table 1).

**Table 1.** Nomenclature.

*d*	diameter of spheres, mm
*D*	tube diameter, mm
*D*p	radial mass dispersion coefficient, cm^2^ s^−1^
*H*	flame height, mm
*L*	packing bed length, mm
*t*	time, s
*u*	mixture velocity in packed bed, m s^−1^
*u*_g_	mixture velocity at inlet, m s^−1^
*V*_CH4_	CH_4_ flow rate, slm
*V*_air_	air flow rate, slm
Greek symbols	
*α*	excess air ratio

Few investigations have been related to diffusion filtration combustion. In the experimental study of a methane–oxygen diffusion flame in a cylindrical packed bed, the flame shapes in the packed beds were constructed from photographs of the upper surface of the packed bed combustor [11]. Kamiuto & Miyamoto [12] developed a flame sheet model that considered the mass dispersion in the packed bed. It was shown that the proposed model can reasonably predict the effects of several parameters on the flame shape.

Dobrego *et al*. [13] used a two-dimensional, multi-component model to simulate non-premixed filtration combustion. The thermal non-equilibrium was accounted for, a one-step kinetics mechanism was used and a parametric study of the radiative efficiency of the combustor was performed. The simulation of non-premixed filtration combustion predicted the high stability of the flame at a low equivalence ratio. Shi *et al*. [14] conducted numerical simulations of diffusion combustion in a plane-parallel packed bed, and their predictions showed that diffusion combustion in a packed bed remains the essential characteristic of an open-space diffusion flame. Ning *et al*. [15] experimentally investigated methane/air diffusion combustion in a Y-shaped meso-scale porous media combustor with three different diameters. They found that the application of porous media can significantly expand the flammable range compared to the conventional one. Shi *et al*. [16] recently reported that two types of flame, the surface flame above the pellets and the immersed flame within the packed bed, coexist in a plane-parallel pellet-packed bed with different bed lengths.

An axial symmetry laminar diffusion flame in an open space has been extensively studied for over 100 years, and significant progress has been made in the theoretical, experimental and numerical study of flame properties [17]. Special attention has been focused on the flame stability, flame shape, soot formation and the role of buoyancy and dilution in the flame properties [18–21]. These developments helped in the design and optimization of practical combustion systems, such as turbines and industrial furnaces, which employ a diffusion flame as the basic combustion element.

As discussed above, little attention has been devoted to the study of diffusion flame structures and characteristics in porous media. Diffusion filtration combustion combines some properties of diffusion and premixed combustion in porous media [13]. Diffusion combustion is an important issue for both the safety and technology of industrial processes. These factors encouraged us to explore diffusion filtration combustion flame properties.

In the present work, we experimentally study the axial symmetry of an over-ventilated CH_4_/air diffusion flame to determine the flame shape, flame height and stability in a packed bed in which a cylindrical fuel stream is surrounded by a coflow air nozzle. After ignition, pellets are poured into the combustor to form a packed bed above the nozzle of the fuel, and then, combustion occurs above the packed bed. The effect of the packed bed length on the flame properties is evaluated.

## 2. Experiments

### 2.1. Experiment apparatus

**Figure 1.**
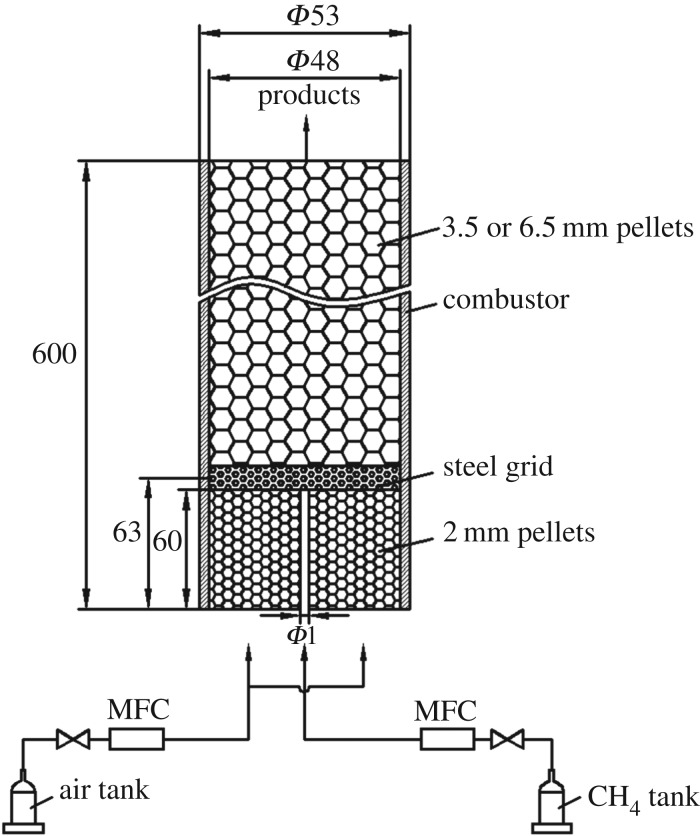
The experimental set-up.

Figure 1 shows the experimental facility in which the diffusion flame structure is measured. It consists of two main components: the combustor (quartz glass tube) packed with alumina pellets and a flow measurement and control system. An air compressor with pressure regulator supplies air to a tank. Both air and CH_4_ are controlled and metered by a mass flow controller.

#### 2.1.1. The combustor

As shown in figure 1, the combustor is a quartz tube with a length of 0.6 m, an internal diameter of *D* = 48 mm and a wall thickness of 2.5 mm. The fuel nozzle is oriented vertically upward on the axis of the cylindrical combustor. Over-ventilated axisymmetric, co-flowing, non-premixed laminar flames are generated in the combustor, which is packed with alumina pellets. Methane flows through a tube with an inner diameter of 1.0 mm (a wall thickness of 0.5 mm), and air flows through an annular region between this tube and a 48 mm diameter concentric tube. To provide the homogeneous mixture, alumina pellets with a diameter of 2.0 mm are packed up to a length of 60 mm between the outer surface of the fuel tube and the quartz glass inner surface, and the packed length is equal to that of the fuel-tube nozzle. The fuel nozzle and the 2.0 mm pellets are covered with a steel grid to form a combustion space above the grid. After ignition, pellets of diameter 3.5 or 6.5 mm are poured into the combustor to increase the bed length up to 20 mm each time. The total length of the packed bed can be up to 240 mm, depending on the experimental condition.

#### 2.1.2. Experimental procedures

The experimental procedures are as follows. Initially, the combustor is kept empty and methane and air are fed in at predetermined values. After ignition, pellets are poured into the combustor and the packed bed length is increased with an increment of 20 mm. The system is left to stabilize for approximately 10 min before any operation is undertaken. Finally, we take photographs from the upper and side views of the combustor using digital video (Sony HDR-PJ30E) and a digital camera (Olympus SZ-30MR). Subsequently, the bed length is increased in steps of 20 mm. The packed bed length varies from 0 to 240 mm. The study is conducted with methane flow rates *V*_CH4_ of 0.5, 0.7 and 0.9 standard litres per minute (slm). The overall excess air ratio *α* is kept constant at 1.2.

## 3. Experimental results and discussion

### 3.1. Flame shapes for different bed lengths

In our experiment, the upper limit of *V*_CH4_ is 0.9 slm to ensure that the combustion is laminar diffusion combustion. The actual flame height can be determined by measuring the flame gas composition [17]. In this study, we assume that the flame height coincides with the height of the visible tip. The flame height is estimated visually, which leads to the overestimation of the flame height compared to the actual flame height. The flame height is an average of two times that of the experimental measurement, and the uncertainty of the flame height is 8%.

**Figure 2.**
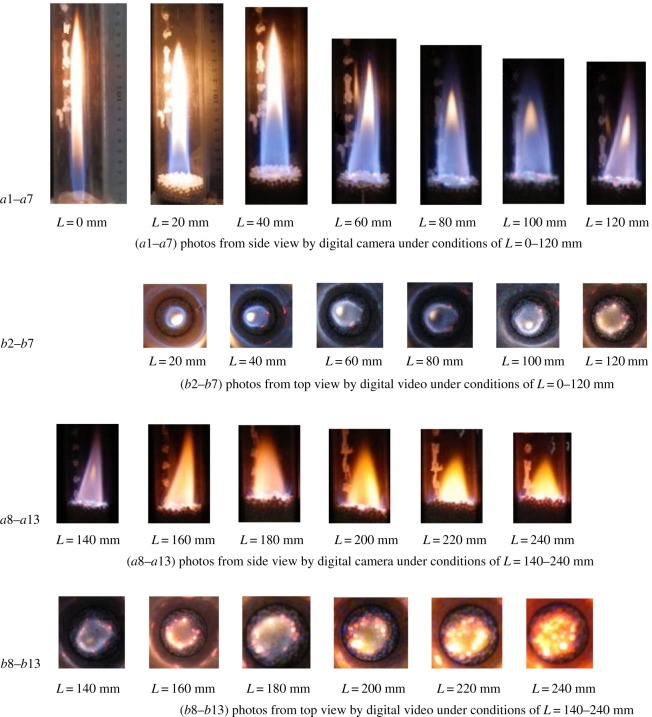
Direct photos of flames at different packing bed length under conditions of *d* = 3.5 mm, *V*_CH4_ = 0.7 slm, *α*=1.2. (*a*1–*a*13) Photos by digital camera from side view; (*b*2–*b*13) photos by digital video from top view.

Figure 2 shows the series of flame shapes as a function of the bed length *L* with the pellet diameter *d* = 3.5 mm and *α* = 1.2. For later analysis, the photos in figure 2 are named in sequence as figure *a*1–*a*13 and *b*2–*b*13, corresponding to digital photos and videos, respectively.

It is found that the combustion occurs above the pellets' surface rather than within the packed bed. Diffusion flames in plane-parallel packed beds were experimentally observed by Kamiuto & Miyamoto [12], who conducted experimental studies on diffusion combustion for CH_4_ + N_2_/O_2_ in plane-parallel packed beds. Their results showed that the flame was immersed in the packed bed. A cylindrical coflow diffusion flame in porous media was numerically verified by Dobrego *et al*. [13]. However, diffusion flames above the pellets’ surface are observed experimentally in our study within the operating parameter range of *d* = 3.5 mm and *V*_CH4_ = 0.5–0.9 slm.

When the quartz glass tube is empty and the length of the packed bed is zero, the typical diffusion flame is observed (figure 2*a*1). The main reaction zone with a blue colour is distinguished from the luminous yellow zone. As shown in figure 2, the flame shape and colour vary with the bed length. If the bed length is increased to 20 mm, the flame becomes much shorter and wider (figure 2*a*2) owing to the gas spreading in sections and dispersing in the porous media. The dispersion of the mixture in the porous media can give rise to increasing radial mass transport, and CH_4_ may exist in this much wider zone. Consequently, it is not surprising that the flame front will become increasingly larger as the bed length increases. Therefore, it is logical that the flame width at the pellets' surface continually increases when *L* ≤ 240 mm.

As shown in figure 2*a*1–*a*8, the closed luminosity yellow zones become shorter when 0 mm ≤ *L* ≤ 140 mm and disappear completely at *L* = 160 mm when increasing the bed length from 20 to 160 mm. As shown in figure 2*a*9–*a*13, if *L* is greater than 140 mm, say from 160 to 240 mm, the colour of the flame will change to yellow. In these cases, the colour may be influenced by the surrounding high temperature, and hence, it may not directly indicate gas combustion, but may reflect the colour of the porous media with a high temperature. As the bed length increases, the fuel and air will mix more effectively, and hence, the combustion on the pellets’ surface is partly or even fully premixed. From figure 2*b*2–*b*13, we see that the colours of the flames are similar to those of figure 2*a*1–2*a*13. These luminous zones with an approximately annular shape at the bed's surface present flame fronts whose sizes increase as the bed length increases from 20 to 240 mm. The annulus-like shape gradually changes to a full circle whose diameter is slightly less than the inner diameter of the quartz glass tube.

An asymmetric flame, or an inclined flame, as it is called, is observed in the experiment. Certainly, we also find the inclined flame in other cases, and the direction of the inclination is not determined (figure 2*a*4, *a*7–*a*9 and *a*13). The inclination may be caused by the random structure of the packed bed rather than the nozzle's inclination because we found no inclination of the flame when there were no pellets in the tube or if the length of the packed bed was small.

### 3.2. Influence of bed length on flame height

**Figure 3.**
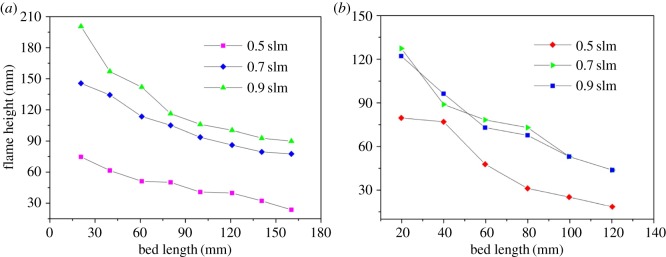
Variation of flame height as a function of bed length for different *V*_CH4_ at *d* = 1 mm, *α*=1.2. (*a*) *d* = 3.5 mm; (*b*) *d* = 6.5 mm.

Figure 3 illustrates the influence of the bed length on the flame height for different values of *V*_CH4_ at *d* = 3.5 and 6.5 mm. As shown in figure 3, the bed length has a significant effect on the flame height. Increasing the bed length leads to a reduction in the flame height. For a constant pellet diameter, an increase in the bed length leads to an increase in the fuel and air residence time in the packed bed. Consequently, fuel may exist on a wider lateral surface before entering the bed surface, and the combustion flame covers a wider range at the bed surface, thus leading to a decrease in flame height.

The radial dispersion coefficient *D*_p_ can be expressed as *D*_p_ =0.1*du*, where *u* is the mixture velocity in the porous media [12]. The influence of the pellet diameter on the flame height in figure 3 can be explained based on the above empirical equation. The smaller the pellet diameter is, the smaller the *D*_p_ that is attained. Therefore, with *u* held constant, the dispersion effect in a packed bed of 3.5 mm pellets is smaller than that in a packed bed of 6.5 mm pellets. This outcome means that the dispersion effect is enhanced for a packed bed filled with pellets of greater diameter. Therefore, as shown in figure 3, with all other conditions fixed, the flame height in a packed bed of 3.5 mm pellets is greater than in a packed bed of 6.5 mm pellets. From figure 3, we can see that the influence of the gas-mixture velocity on the flame height is similar to that of classical laminar diffusion combustion. In general, an increase in the gas-mixture velocity leads to an increase in the flame height. However, for *d* = 6.5 mm, when *u*_g_ is increased to 0.9 slm, the flame heights are almost the same as when *u*_g_ = 0.7 slm, perhaps because unstable combustion occurs with the pellets of greater diameter that are used in the experiment. We will discuss this issue in the following section.

### 3.3. Multiple combustion zones and oscillatory flame

**Figure 4.**
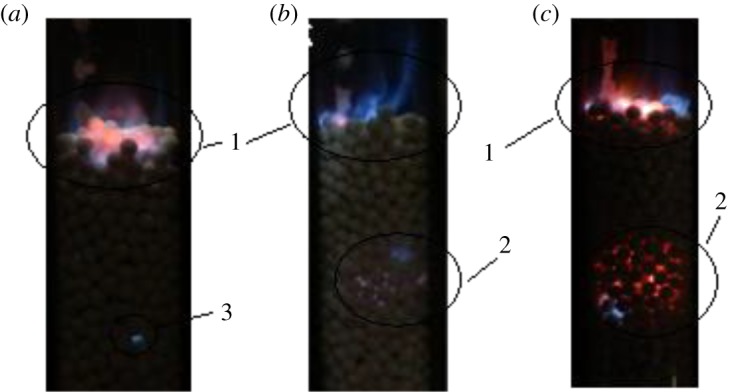
Direct photography of flame oscillatory. (*a*) *L* = 140 mm, *V*_CH4_ = 0.5 slm, *α* = 1.2. (*b*) *L* = 240 mm, *V*_CH4_ = 0.7 slm, *α* = 1.2. (*c*) *L* = 240 mm, *V*_CH4_ = 0.9 slm, *α* = 1.2. 1, main reaction. 2, oscillatory combustion. 3, isolated reaction zone.

Multiple combustion zones in the packed bed are observed experimentally under the condition of *d* = 6.5 mm when the bed length is greater than a certain value. As shown in figure 4*a*, a very stable reaction zone with relatively weak luminosity separated from the main reaction zone occurs on the upstream side of the main reaction zone. This flame appears to be small in size, but it is very stable and extinguished until oscillatory combustion occurs on the upstream side of the main reaction zone.

As shown in figure 4*b,c*, oscillatory combustion zones are observed under conditions of *V*_CH4_ = 0.7 slm and *V*_CH4_ = 0.9 slm at *L* = 240 mm. Oscillatory combustion zones occur on the upstream side when the length of the packed bed exceeds a certain value. The flame ignites and extinguishes with high frequency followed by the pulsing voice of some few hertz frequency. The flame gradually becomes dark, extinguishing after an hour of oscillation. Again, if the combustion is ignited from the top of the bed, the oscillation can continue for 1 h, and then combustion stops. This outcome may be explained as follows. In the random structure of a packed bed with 5.6 mm pellets, there exists a local porosity that is greater than the average, and this helps to generate a combustion space where the stoichiometric ratio exists. As shown in figure 4*c*, in the middle of the oscillatory combustion zone, there may be a zone of high solid temperature that acts as a fixed ‘external’ heat source. The gas mixtures are ignited near the high-temperature zone, and a flame front propagates downstream until it reaches the point of extinction. There may be another explanation for the oscillation. There is some fuel at the surface of the bed that is not consumed completely. After ignition on the surface, the flame will penetrate the packed bed and approach the place where the stoichiometric ratio exists. However, the combustion at this position cannot be stabilized, and a quench occurs. It is interesting that the quench will lead to a contribution of fuel that is not consumed completely again on the top surface; this fuel will be ignited, and the same routine will occur. Then, the oscillation arises continually.

Surface combustion on the pellet surfaces is influenced by the oscillatory motion of the unstable combustion in position 2. Its flame front with the blue colour at the inner surface oscillates at the same frequency as position 2. This similar phenomenon was detected in a previous experiment [12]. Kamiuto & Miyamoto [12] reported that diffusion combustion in porous media was always followed by pulsative sounds of a few hertz. They suggested that a flame propagates within the packed bed from the upper surface of the combustor towards the region upstream along the flame sheet and is extinguished at the exit of the combustor.

## 4. Conclusion

The experimental facility consists of the combustor packed with alumina pellets, a flow measurement and a control system. We conducted experimental studies on diffusion combustion with respect to flame height, flame shape and its ability to use a methane–air mixture. The flame is generally stable for the smaller pellet diameter of 3.5 mm, and the bed length has a significant effect on the flame shape and height. The shape of these flames is similar to that of conventional diffusion flames when the packed bed length is smaller. The flame above the pellets' surface becomes wider and shorter as the bed length increases. The changed colour indicates that the non-premixed combustion transfers to partially premixed combustion, and even premixed combustion, as the bed length increases. However, when the pellet diameter increases to 6.5 mm, unstable combustion occurs for greater bed lengths. We observe three different flame characteristics: a stably isolated combustion zone, an oscillatory combustion zone and surface combustion. The reason for oscillatory combustion on the upstream side of the main reaction zone may be that there exists a local porosity that is greater than the average of the random structure of a packed bed with 5.6 mm pellets, and this helps to generate a combustion space where the stoichiometric ratio exists. There may be another explanation for the oscillation. Some fuel at the surface of the bed may not be consumed completely, in which case the flame will penetrate the packed bed and approach the place where the stoichiometric ratio exists.

## Data accessibility

Our data are deposited at Dryad: http://dx.doi.org/10.5061/dryad.1fv8372 [22].
